# 3-Hydr­oxy-1,2,3,9-tetra­hydro­pyrrolo[2,1-*b*]quinazolin-4-ium chloride dihydrate: (+)-vasicinol hydro­chloride dihydrate from *Peganum harmala* L

**DOI:** 10.1107/S1600536809003766

**Published:** 2009-02-06

**Authors:** Amir Muhammad Khan, Ghulam Abbas, Rizwana Aleem Qureshi, Uzma Khan, Muhammad Asad Ghufran, Helen Stoeckli-Evans

**Affiliations:** a Department of Plant Sciences, Quaid-i-Azam University, Islamabad, Pakistan; b Well Department Services, Oil and Gas Development Company Ltd, Islamabad, Pakistan; c Institute of Physics, University of Neuchâtel, Rue Emile-Argand 11, CH-2009 Neuchâtel, Switzerland

## Abstract

The title compound, C_11_H_13_N_2_O^+^·Cl^−^·2H_2_O, the dihydrate of (+)-vasicinol hydro­chloride, is a pyrrolidinoquinazoline alkaloid. It was isolated from the ethyl acetate fraction of the leaves of *Peganum harmala* L. The pyrrolidine ring has an envelope conformation with the C atom at position 2 acting as the flap and the C atom at position 3, carrying the hydroxyl substituent, has an *S* configuration. The absolute configuration was determined as a result of the anomalous scattering of the Cl atom. In the crystal structure, mol­ecules stack along the *a* axis, connected to one another *via* inter­molecular O—H⋯Cl and N—H⋯Cl hydrogen bonds, forming approximately triangular-shaped *R*_2_^1^(7) rings, and O—H⋯Cl and O—H⋯O hydrogen bonds, forming penta­gonal-shaped *R*_5_^4^(10) rings. The overall effect is a ribbon-like arrangement running parallel to the *a* axis.

## Related literature

For the isolation (+)-vasicinol and the crystal structure analysis of (+)-vasicinol hydro­bromide, see: Joshi *et al.* (1996 ▶). For general background on pyrrolidino-quinazoline alkaloids and their structures, see: Szulzewsky *et al.* (1976 ▶): Openshaw (1953 ▶); Bailey (1986 ▶); Rizk (1986 ▶); Tashkhodzhaev *et al.* (1995 ▶); Turgunov *et al.* (1995 ▶). For a study on the anti-Leishmaniasis activity of (+)-vasicinol hydro­chloride dihydrate, see: Misra *et al.* (2008 ▶). For further related literature on natural products, see: Hilal & Youngken (1983 ▶); Mirzakhmedov *et al.* (1975 ▶). For hydrogen-bond motifs, see: Bernstein *et al.* (1995 ▶). For puckering parameters, see: Cremer & Pople (1975 ▶).
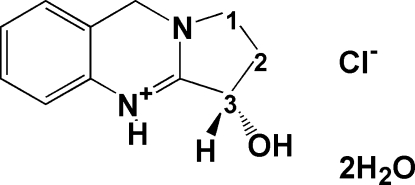


## Experimental

### 

#### Crystal data

C_11_H_13_N_2_O^+^·Cl^−^·2H_2_O
*M* *_r_* = 260.72
Orthorhombic, 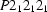

*a* = 7.0386 (6) Å
*b* = 9.5752 (10) Å
*c* = 18.4041 (18) Å
*V* = 1240.4 (2) Å^3^
*Z* = 4
Mo *K*α radiation
μ = 0.31 mm^−1^
*T* = 173 (2) K
0.42 × 0.19 × 0.11 mm

#### Data collection

Stoe IPDS diffractometer
Absorption correction: none
8680 measured reflections
2422 independent reflections
1834 reflections with *I* > 2σ(*I*)
*R*_int_ = 0.034

#### Refinement

*R*[*F* ^2^ > 2σ(*F* ^2^)] = 0.029
*wR*(*F* ^2^) = 0.061
*S* = 0.90
2422 reflections
216 parameters
2 restraints
All H-atom parameters refined
Δρ_max_ = 0.20 e Å^−3^
Δρ_min_ = −0.14 e Å^−3^
Absolute structure: Flack (1983 ▶), 984 Friedel pairs
Flack parameter: 0.004 (64)

### 

Data collection: *EXPOSE* in *IPDS-I* (Stoe & Cie, 2000 ▶); cell refinement: *CELL* in *IPDS-I*; data reduction: *INTEGRATE* in *IPDS-I*; program(s) used to solve structure: *SHELXS97* (Sheldrick, 2008 ▶); program(s) used to refine structure: *SHELXL97* (Sheldrick, 2008 ▶); molecular graphics: *ORTEP-3* (Farrugia, 1997 ▶) and *Mercury* (Macrae *et al.*, 2006 ▶); software used to prepare material for publication: *SHELXL97*.

## Supplementary Material

Crystal structure: contains datablocks I, global. DOI: 10.1107/S1600536809003766/lh2765sup1.cif

Structure factors: contains datablocks I. DOI: 10.1107/S1600536809003766/lh2765Isup2.hkl

Additional supplementary materials:  crystallographic information; 3D view; checkCIF report

## Figures and Tables

**Table 1:** Hydrogen-bond geometry (Å, °)

*D*—H⋯*A*	*D*—H	H⋯*A*	*D*⋯*A*	*D*—H⋯*A*
O1—H1*O*⋯Cl1^i^	0.90 (2)	2.20 (2)	3.086 (2)	171 (2)
N9—H9*N*⋯Cl1^i^	0.83 (2)	2.35 (2)	3.155 (2)	167 (2)
O1*W*—H1*WA*⋯Cl1^ii^	0.83 (4)	2.39 (4)	3.204 (2)	167 (3)
O1*W*—H1*WB*⋯O2*W*^ii^	0.79 (4)	1.96 (4)	2.720 (3)	162 (4)
O2*W*—H2*WA*⋯Cl1^iii^	0.83 (4)	2.35 (4)	3.173 (2)	175 (3)
O2*W*—H2*WB*⋯O1*W*^iv^	0.89 (3)	1.83 (3)	2.718 (3)	179 (5)

Symmetry codes: (i) 
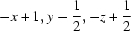
; (ii) 
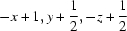
; (iii) 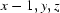; (iv) 
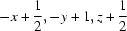
.

**Fig. 1.**
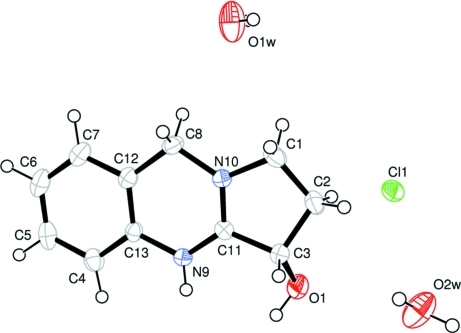
The molecular structure of the title compound, showing the atomic numbering scheme and displacement ellipsoids drawn at the 50% probability level.

**Fig. 2.**
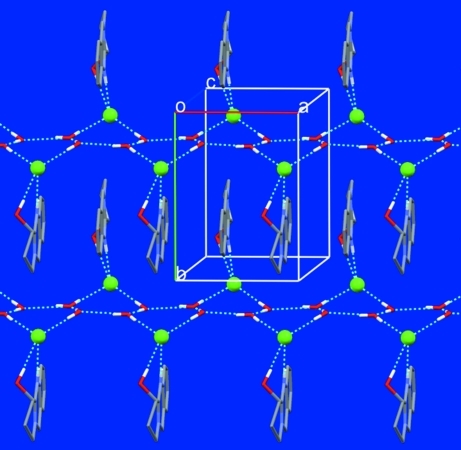
View along the c direction of the crystal packing of the title compound, showing the formation of the triangular-shaped [R12(7)], and pentagonal-shaped [R54(10)] rings of hydrogen bonds (dashed blue lines; Cl- green ball; H-atoms not involved in hydrogen bonding have been removed for clarity).

**Fig. 3.**
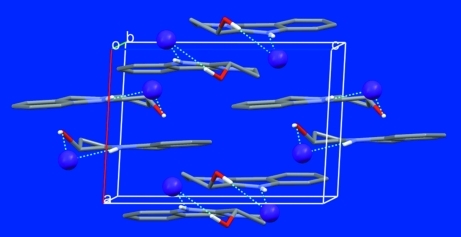
View along the a axis of the crystal packing of (+)-Vasicinol hydrobromide (Joshi et al., 1996), showing the formation of the trianglular-shaped [R12(7)] rings of hydrogen bonds [Br- purple ball; H-atoms not involved in hydrogen bonding have been removed for clarity].

## supplementary crystallographic information

### Comment

*Peganum harmala* L is a member of the family Zygophyllaceae (Hilal and Youngken, 1983). This plant is commonly distributed in the Attock District, Islamabad, including the Margalla Hills. Several alkaloids have been isolated from the seeds and roots of this plant and have been identified as chemicals with a β-carboline structure, such as harmine, harmaline, harmalol, and harman (Bailey, 1986; Rizk, 1986), or with a quinazoline structure, such as vasicine and vasicinon (Openshaw, 1953; Bailey, 1986; Joshi *et al.*, 1996). Here we report on the crystal structure of the title compound, the dihydrate of (+)Vasicinol hydrochloride. It is a pyrrolidino-quinazoline alkaloid and was isolated from the ethylacetate fraction of the leaves of *Peganum harmala* L, collected from the Margalla Hills, Islamabad.

The molecular structure of the title compound is shown in Fig. 1. Dimensions are similar to those observed in other pyrrolidino-quinazoline alkaloids (Joshi *et al.*, 1996; Tashkhodzhaev *et al.*, 1995; Turgunov *et al.*, 1995; Szulzewsky *et al.*, 1976). The title compound crystallizes in the non-centrosymmetric orthorhombic space group *P*2_1_2_1_2_1_. The crystal structure of (+)-Vasicinol hydrobromide has been reported previously (Joshi *et al.*, 1996), and crystallizes in the non-centrosymmetric monoclinic space group *P*2_1_, with two independent molecules per asymmetric unit.

The pyrrolidine ring has an envelope conformation on atom C2; the puckering parameters (Cremer & Pople, 1975) are Q(2) = 0.243 (2) Å, and φ(2) = 253.8 (5)°. The carbon atom carrying the hydroxyl substituent, atom C3, has a S-configuration. The absolute configuration of the title compound was determined as a result of the anomalous scattering of the Cl-atom.

In the crystal the molecules stacks up the *a* axis and are connected to one another *via* a series of O—H···Cl, N—H···Cl hydrogen bonds, with approximately triangular-shaped *R*^1^_2_(7) rings (Bernstein *et al.*,  1995) and O—H···Cl and O—H···O hydrogen bonds, forming pentagonal-shaped *R*_5_^4^(10) rings [see Fig. 2 and Table 1]. In this way a ribon-like arrangement is formed running parallel to the *a* axis. The same triangular arrangement, involving O—H···halide^-^ and N—H···halide^-^ hydrogen bonds, is also observed in the crystal packing of (+)-Vasicine hydrobromide (Fig. 3; Joshi *et al.*, 1996).

### Experimental

*Peganum harmala* L is a member of the family Zygophyllaceae (Hilal & Youngken, 1983). This plant is commonly distributed in the Attock District, Islamabad, including the Margalla Hills, Gilgit: Chilas, Yasin, Gupis, Phunder, Hunza, Skardu. The leaves of the plant were collected from the Margalla Hills, Islamabad and the sample was deposited in the herbarium of Quaid-i-Azam University (ISL) under the accession No. 123774 and 123775. The air dried leaves were powdered and extracted three times with methanol. The extracts were and concentrated to a semi-solid mass. Water was added to this semi-solid mass to form a paste. The extract was then fractioned using different solvents according to their increasing polarity. The title compound was isolated from the ethylacetate fraction of the leaves of *Peganum harmala L* as colourless rod-like crystals.

### Refinement

The absolute configuration of the title compound was determined as a result of the anomalous scattering of the Cl-atom: Flack *x* parameter = -0.0044 with e.s.d. 0.0644; Hooft *y* Parameter Value = 0.0024 with e.s.d. 0.0478. The hydrogen atoms were located in difference Fourier maps. The C-bound H-atoms were freely refined: C—H = 0.92 (2) - 1.04 (2) Å. The O—H and N—H bond distances were restrained to 0.87 (2) and 0.82 (2) %A, respectively: O—H = 0.89 (2) Å and N—H = 0.821 (17) Å. The water H-atoms were refined with *U*_iso_(H) = 1.5*U*_eq_(O): O—H = 0.79 (4) - 0.89 (3) Å.

### Crystal data

**Table:**

C_11_H_13_N_2_O^+^·Cl^−^·2H_2_O	*F*(000) = 552
*M**_r_* = 260.72	*D*_x_ = 1.396 Mg m^−^^3^
Orthorhombic, *P*2_1_2_1_2_1_	Mo *K*α radiation, λ = 0.71073 Å
Hall symbol: P 2ac 2ab	Cell parameters from 6244 reflections
*a* = 7.0386 (6) Å	θ = 2.2–25.9°
*b* = 9.5752 (10) Å	µ = 0.31 mm^−^^1^
*c* = 18.4041 (18) Å	*T* = 173 K
*V* = 1240.4 (2) Å^3^	Rod, colourless
*Z* = 4	0.42 × 0.19 × 0.11 mm

### Data collection

**Table:**

Stoe IPDS diffractometer	1834 reflections with *I* > 2σ(*I*)
Radiation source: fine-focus sealed tube	*R*_int_ = 0.034
graphite	θ_max_ = 26.1°, θ_min_ = 2.2°
phi rotation scans	*h* = −8→8
8680 measured reflections	*k* = −11→11
2422 independent reflections	*l* = −22→22

### Refinement

**Table:**

Refinement on *F*^2^	Secondary atom site location: difference Fourier map
Least-squares matrix: full	Hydrogen site location: inferred from neighbouring sites
*R*[*F*^2^ > 2σ(*F*^2^)] = 0.029	All H-atom parameters refined
*wR*(*F*^2^) = 0.061	*w* = 1/[σ^2^(*F*_o_^2^) + (0.033*P*)^2^] where *P* = (*F*_o_^2^ + 2*F*_c_^2^)/3
*S* = 0.90	(Δ/σ)_max_ < 0.001
2422 reflections	Δρ_max_ = 0.20 e Å^−^^3^
216 parameters	Δρ_min_ = −0.14 e Å^−^^3^
2 restraints	Absolute structure: Flack (1983), 984 Friedel pairs
Primary atom site location: structure-invariant direct methods	Flack parameter: 0.004 (64)

### Special details

**Table:**

Geometry. Bond distances, angles *etc*. have been calculated using the rounded fractional coordinates. All su's are estimated from the variances of the (full) variance-covariance matrix. The cell e.s.d.'s are taken into account in the estimation of distances, angles and torsion angles
Refinement. Refinement of *F*^2^ against ALL reflections. The weighted *R*-factor *wR* and goodness of fit *S* are based on *F*^2^, conventional *R*-factors *R* are based on *F*, with *F* set to zero for negative *F*^2^. The threshold expression of *F*^2^ > σ(*F*^2^) is used only for calculating *R*-factors(gt) *etc*. and is not relevant to the choice of reflections for refinement. *R*-factors based on *F*^2^ are statistically about twice as large as those based on *F*, and *R*- factors based on ALL data will be even larger.

### Fractional atomic coordinates and isotropic or equivalent isotropic displacement parameters (Å^2^)

**Table:**

	*x*	*y*	*z*	*U*_iso_*/*U*_eq_	
O1	0.3510 (2)	0.15059 (15)	0.24928 (8)	0.0409 (5)	
N9	0.2235 (3)	0.16767 (15)	0.09484 (8)	0.0249 (5)	
N10	0.2348 (2)	0.40126 (14)	0.12323 (8)	0.0246 (5)	
C1	0.2373 (4)	0.4955 (2)	0.18629 (12)	0.0348 (8)	
C2	0.2861 (4)	0.3982 (2)	0.24882 (11)	0.0348 (7)	
C3	0.2264 (4)	0.2536 (2)	0.22362 (10)	0.0280 (7)	
C4	0.2242 (3)	0.0847 (2)	−0.02920 (11)	0.0310 (7)	
C5	0.2242 (3)	0.1121 (3)	−0.10295 (10)	0.0373 (7)	
C6	0.2232 (4)	0.2484 (3)	−0.12768 (11)	0.0398 (8)	
C7	0.2228 (3)	0.3570 (2)	−0.07828 (10)	0.0339 (7)	
C8	0.2231 (4)	0.45209 (19)	0.04834 (10)	0.0287 (7)	
C11	0.2303 (3)	0.27062 (18)	0.14208 (9)	0.0223 (6)	
C12	0.2237 (3)	0.33226 (19)	−0.00429 (9)	0.0253 (6)	
C13	0.2242 (3)	0.19435 (18)	0.01937 (9)	0.0227 (6)	
O1W	0.4964 (3)	0.7633 (3)	0.06429 (11)	0.0639 (8)	
O2W	0.1191 (3)	0.2477 (2)	0.42346 (11)	0.0598 (8)	
Cl1	0.80565 (8)	0.38150 (5)	0.32092 (3)	0.0358 (2)	
H1A	0.329 (3)	0.563 (2)	0.1789 (12)	0.033 (6)*	
H1B	0.113 (4)	0.540 (2)	0.1877 (14)	0.049 (7)*	
H1O	0.298 (4)	0.071 (2)	0.2333 (13)	0.0610*	
H2A	0.225 (4)	0.422 (2)	0.2921 (12)	0.038 (6)*	
H2B	0.431 (3)	0.395 (2)	0.2595 (11)	0.040 (6)*	
H3	0.095 (3)	0.231 (2)	0.2387 (11)	0.036 (6)*	
H4	0.233 (3)	−0.012 (2)	−0.0084 (10)	0.034 (6)*	
H5	0.222 (4)	0.034 (2)	−0.1392 (12)	0.053 (7)*	
H6	0.224 (3)	0.266 (2)	−0.1810 (13)	0.047 (6)*	
H7	0.217 (4)	0.454 (2)	−0.0961 (12)	0.051 (7)*	
H8A	0.108 (3)	0.502 (2)	0.0441 (11)	0.029 (6)*	
H8B	0.331 (3)	0.517 (2)	0.0407 (11)	0.033 (6)*	
H9N	0.211 (4)	0.0872 (17)	0.1102 (11)	0.0370*	
H1WA	0.432 (5)	0.792 (4)	0.099 (2)	0.0960*	
H1WB	0.603 (5)	0.764 (4)	0.077 (2)	0.0960*	
H2WA	0.033 (5)	0.282 (4)	0.3988 (19)	0.0900*	
H2WB	0.081 (5)	0.245 (4)	0.4695 (18)	0.0900*	

### Atomic displacement parameters (Å^2^)

**Table:**

	*U*^11^	*U*^22^	*U*^33^	*U*^12^	*U*^13^	*U*^23^
O1	0.0538 (11)	0.0371 (9)	0.0319 (8)	0.0022 (7)	−0.0108 (7)	0.0022 (7)
N9	0.0323 (11)	0.0191 (7)	0.0233 (8)	−0.0015 (8)	−0.0018 (8)	0.0007 (7)
N10	0.0296 (11)	0.0206 (8)	0.0235 (7)	0.0004 (8)	−0.0027 (7)	0.0010 (6)
C1	0.0445 (17)	0.0241 (10)	0.0357 (11)	−0.0014 (10)	0.0015 (12)	−0.0078 (9)
C2	0.0439 (15)	0.0338 (10)	0.0268 (10)	−0.0057 (12)	0.0001 (11)	−0.0084 (9)
C3	0.0337 (16)	0.0272 (10)	0.0230 (10)	−0.0018 (10)	0.0003 (9)	0.0000 (8)
C4	0.0307 (14)	0.0319 (11)	0.0305 (10)	0.0038 (9)	−0.0018 (10)	−0.0054 (8)
C5	0.0312 (14)	0.0536 (12)	0.0272 (10)	0.0024 (13)	−0.0031 (9)	−0.0119 (11)
C6	0.0360 (15)	0.0600 (14)	0.0234 (10)	0.0003 (13)	−0.0011 (11)	0.0050 (10)
C7	0.0318 (13)	0.0429 (13)	0.0270 (10)	0.0017 (11)	−0.0002 (9)	0.0090 (9)
C8	0.0317 (14)	0.0253 (10)	0.0292 (11)	−0.0001 (11)	−0.0030 (10)	0.0075 (8)
C11	0.0194 (13)	0.0225 (9)	0.0249 (9)	0.0014 (8)	0.0003 (8)	−0.0017 (8)
C12	0.0182 (12)	0.0323 (9)	0.0253 (9)	0.0002 (9)	−0.0014 (9)	0.0027 (8)
C13	0.0199 (12)	0.0281 (9)	0.0200 (9)	−0.0006 (8)	−0.0001 (8)	0.0004 (7)
O1W	0.0428 (12)	0.1035 (16)	0.0453 (11)	0.0043 (12)	0.0030 (9)	−0.0184 (11)
O2W	0.0432 (13)	0.0885 (15)	0.0477 (11)	0.0093 (11)	0.0014 (9)	0.0226 (11)
Cl1	0.0414 (3)	0.0279 (2)	0.0380 (3)	0.0034 (2)	0.0001 (3)	−0.0046 (2)

### Geometric parameters (Å, °)

**Table:**

O1—C3	1.402 (3)	C5—C6	1.382 (4)
O1—H1O	0.90 (2)	C6—C7	1.381 (3)
O1W—H1WB	0.79 (4)	C7—C12	1.382 (2)
O1W—H1WA	0.83 (4)	C8—C12	1.502 (3)
O2W—H2WA	0.83 (4)	C12—C13	1.391 (2)
O2W—H2WB	0.89 (3)	C1—H1B	0.97 (3)
N9—C13	1.412 (2)	C1—H1A	0.92 (2)
N9—C11	1.315 (2)	C2—H2A	0.93 (2)
N10—C11	1.299 (2)	C2—H2B	1.04 (2)
N10—C8	1.464 (2)	C3—H3	0.99 (2)
N10—C1	1.470 (3)	C4—H4	1.004 (19)
N9—H9N	0.825 (17)	C5—H5	1.00 (2)
C1—C2	1.520 (3)	C6—H6	1.00 (2)
C2—C3	1.519 (3)	C7—H7	0.99 (2)
C3—C11	1.510 (2)	C8—H8A	0.94 (2)
C4—C5	1.382 (3)	C8—H8B	0.99 (2)
C4—C13	1.379 (3)		
			
C3—O1—H1O	103.1 (16)	C4—C13—C12	121.34 (16)
H1WA—O1W—H1WB	107 (4)	N10—C1—H1A	108.8 (14)
H2WA—O2W—H2WB	108 (3)	C2—C1—H1B	116.8 (15)
C11—N9—C13	120.96 (15)	N10—C1—H1B	106.2 (14)
C1—N10—C11	112.38 (15)	C2—C1—H1A	112.5 (13)
C1—N10—C8	122.67 (14)	H1A—C1—H1B	109.0 (18)
C8—N10—C11	124.78 (15)	C1—C2—H2B	112.5 (11)
C13—N9—H9N	120.4 (14)	C3—C2—H2A	110.8 (13)
C11—N9—H9N	118.5 (14)	H2A—C2—H2B	107 (2)
N10—C1—C2	102.95 (15)	C3—C2—H2B	107.6 (11)
C1—C2—C3	105.36 (17)	C1—C2—H2A	113.1 (13)
O1—C3—C2	111.4 (2)	O1—C3—H3	109.7 (12)
O1—C3—C11	113.54 (17)	C11—C3—H3	108.6 (12)
C2—C3—C11	101.54 (15)	C2—C3—H3	111.9 (11)
C5—C4—C13	119.47 (19)	C13—C4—H4	117.1 (11)
C4—C5—C6	120.2 (2)	C5—C4—H4	123.3 (11)
C5—C6—C7	119.61 (19)	C4—C5—H5	120.8 (12)
C6—C7—C12	121.29 (19)	C6—C5—H5	119.0 (12)
N10—C8—C12	110.68 (14)	C7—C6—H6	121.4 (11)
N9—C11—C3	125.13 (16)	C5—C6—H6	119.0 (11)
N10—C11—C3	111.72 (15)	C12—C7—H7	119.3 (13)
N9—C11—N10	123.11 (16)	C6—C7—H7	119.4 (13)
C8—C12—C13	121.58 (15)	N10—C8—H8B	107.4 (12)
C7—C12—C8	120.30 (16)	N10—C8—H8A	107.1 (12)
C7—C12—C13	118.12 (16)	H8A—C8—H8B	109.2 (17)
N9—C13—C4	119.99 (16)	C12—C8—H8A	109.6 (12)
N9—C13—C12	118.67 (15)	C12—C8—H8B	112.7 (12)
			
C13—N9—C11—N10	1.2 (3)	O1—C3—C11—N10	−135.24 (18)
C13—N9—C11—C3	178.7 (2)	C2—C3—C11—N9	166.7 (2)
C11—N9—C13—C4	177.6 (2)	C2—C3—C11—N10	−15.5 (3)
C11—N9—C13—C12	−2.8 (3)	C13—C4—C5—C6	−0.3 (3)
C8—N10—C1—C2	−170.1 (2)	C5—C4—C13—N9	179.8 (2)
C11—N10—C1—C2	14.5 (3)	C5—C4—C13—C12	0.2 (3)
C1—N10—C8—C12	179.9 (2)	C4—C5—C6—C7	0.2 (4)
C11—N10—C8—C12	−5.3 (3)	C5—C6—C7—C12	0.1 (4)
C1—N10—C11—N9	178.6 (2)	C6—C7—C12—C8	−180.0 (2)
C1—N10—C11—C3	0.7 (3)	C6—C7—C12—C13	−0.3 (3)
C8—N10—C11—N9	3.3 (3)	N10—C8—C12—C7	−176.89 (19)
C8—N10—C11—C3	−174.5 (2)	N10—C8—C12—C13	3.4 (3)
N10—C1—C2—C3	−23.3 (3)	C7—C12—C13—N9	−179.5 (2)
C1—C2—C3—O1	144.52 (19)	C7—C12—C13—C4	0.1 (3)
C1—C2—C3—C11	23.3 (3)	C8—C12—C13—N9	0.2 (3)
O1—C3—C11—N9	47.0 (3)	C8—C12—C13—C4	179.8 (2)

### Hydrogen-bond geometry (Å, °)

**Table:**

*D*—H···*A*	*D*—H	H···*A*	*D*···*A*	*D*—H···*A*
O1—H1O···Cl1^i^	0.90 (2)	2.20 (2)	3.086 (2)	171 (2)
N9—H9N···Cl1^i^	0.83 (2)	2.35 (2)	3.155 (2)	167 (2)
O1W—H1WA···Cl1^ii^	0.83 (4)	2.39 (4)	3.204 (2)	167 (3)
O1W—H1WB···O2W^ii^	0.79 (4)	1.96 (4)	2.720 (3)	162 (4)
O2W—H2WA···Cl1^iii^	0.83 (4)	2.35 (4)	3.173 (2)	175 (3)
O2W—H2WB···O1W^iv^	0.89 (3)	1.83 (3)	2.718 (3)	179 (5)

Symmetry codes: (i) −*x*+1, *y*−1/2, −*z*+1/2; (ii) −*x*+1, *y*+1/2, −*z*+1/2; (iii) *x*−1, *y*, *z*; (iv) −*x*+1/2, −*y*+1, *z*+1/2.
